# Supplementary material for: Bleeding, cramping, and satisfaction among new copper IUD users: A prospective study
Source: PLoS One. 2018 Nov 7;13(11):e0199724. doi: 10.1371/journal.pone.0199724 (PMC6221252; doi:10.1371/journal.pone.0199724)
Supplement: S1 Supporting Information — Table A: Structural equation model parameter estimates for growth curves of bleeding (PBAC Score); Unimputed, complete case analysis. Table B: Structural equation model parameter estimates for growth curves of bleeding (PBAC Score); Multiple imputation, 30 imputations. Table C: Structural equation model parameter estimates for growth curves of IUD Satisfaction (5-level Likert scale); Unimputed, complete case analysis. Table D. Structural equation model parameter estimates for growth curves of IUD Satisfaction (5-level ordinal Likert scale); Multiple imputation, 30 imputations. Table E. Structural equation model parameter estimates for growth curves of cramping (6-level ordinal MSQ item); Unimputed, complete case analysis. Table F. Structural equation model parameter estimates for growth curves of cramping (6-level ordinal MSQ item); Multiple imputation, 30 imputations. Table G. IUD Satisfaction predicted by bleeding during study and cramping; Multiple imputation, 30 imputations. Table H. SEM parameter estimates for growth curve trajectories for bleeding including the three retrospective baseline bleeding reports. Fig I. Time series plots summarizing distributions, and predicted mean growth curves of bleeding as measured by the Pictorial Blood Assessment Chart (PBAC), over study month, including the three retrospective baseline bleeding reports (i.e., months 1–3). (DOCX) [file pone.0199724.s001.docx]

**Supporting Information**

For the manuscript:

Bleeding, cramping, and contraceptive method satisfaction among new copper IUD users: A prospective study

Jessica N Sanders^1¶^, Daniel E Adkins^2,3,¶,^*, Simranvir Kaur^4^, Kathryn Storck^1^, Lori M Gawron^1^, David K Turok^1^

^1^ Department of Obstetrics and Gynecology, University of Utah, Salt Lake City, Utah, United States of America

^2^ Department of Sociology, University of Utah, Salt Lake City, Utah, United States of America

^3^ Department of Psychiatry, University of Utah, Salt Lake City, Utah, United States of America

^4^ School of Medicine, University of Utah, Salt Lake City, Utah, United States of America

* Corresponding author

E-mail: daniel.adkins@soc.utah.edu

^¶^ These authors contributed equally to this work.

| **Table A. Structural equation model parameter estimates for growth curves of bleeding (PBAC Score); Unimputed, complete case analysis** | | | |
| --- | --- | --- | --- |
|  | Bleeding | Bleeding | Bleeding |
| Link Function | Linear | Poisson | Negative Binomial |
| Time (study month) | -8.900* | -0.044*** | -0.060** |
|  | (-2.354) | (-3.584) | (-2.643) |
| Intercept | 187.393*** | 5.057*** | 5.126*** |
|  | (13.520) | (66.881) | (56.584) |
| Random intercept variance | 5824.760*** | 0.437*** | 0.312*** |
|  | (3.774) | (6.135) | (4.245) |
| Residual variance | 16514.663*** |  |  |
|  | (12.896) |  |  |
| Overdispersion (ln(α)) |  |  | -0.596*** |
|  |  |  | (-7.579) |
| N | 415 | 415 | 415 |
| AIC | 5296.911 | 5216.148 | 4995.313 |
| BIC | 5313.024 | 5228.233 | 5011.426 |
| t statistics in parentheses |  |  |  |
| * p<0.05; ** p<0.01; *** p<0.001 | |  |  |

| **Table B. Structural equation model parameter estimates for growth curves of bleeding (PBAC Score); Multiple imputation, 30 imputations** | | | |
| --- | --- | --- | --- |
|  | Bleeding | Bleeding | Bleeding |
| Link Function | Linear | Poisson | Negative Binomial |
| Time (study month) | -6.594* | -0.039* | -0.055* |
|  | (-2.222) | (-2.030) | (-2.384) |
| Intercept | 189.572*** | 5.042*** | 5.126*** |
|  | (9.916) | (59.324) | (54.695) |
| Random intercept variance | 11371.852 | 0.460*** | 0.361*** |
|  | (1.193) | (5.601) | (4.210) |
| Residual variance | 25718.860 |  |  |
|  | (1.118) |  |  |
| Overdispersion (ln(α)) |  |  | -0.620*** |
|  |  |  | (-8.039) |
| N_j_ (Assessments) | 462 | 462 | 462 |
| N_i_ (Subjects) | 77 | 77 | 77 |
| t statistics in parentheses |  |  |  |
| * p<0.05; ** p<0.01; *** p<0.001 | |  |  |

| **Table C. Structural equation model parameter estimates for growth curves of IUD Satisfaction (5-level Likert scale); Unimputed, complete case analysis** | | | |
| --- | --- | --- | --- |
|  | IUD Satisfaction | IUD Satisfaction | IUD Satisfaction |
| Link Function | Linear | Ordered logistic | Ordered probit |
| Time (study month) | 0.085** | 0.196** | 0.126*** |
|  | (3.217) | (3.234) | (3.682) |
| Intercept | 3.632*** |  |  |
|  | (34.828) |  |  |
| Random intercept variance | 0.396*** | 3.235*** | 0.842*** |
|  | (4.285) | (3.968) | (3.949) |
| Residual variance | 0.788*** |  |  |
|  | (12.804) |  |  |
| Threshold 1 |  | -3.560*** | -1.778*** |
|  |  | (-9.665) | (-10.060) |
| Threshold 2 |  | -2.563*** | -1.307*** |
|  |  | (-7.910) | (-7.944) |
| Threshold 3 |  | -1.055*** | -0.526*** |
|  |  | (-3.693) | (-3.473) |
| Threshold 4 |  | 1.722*** | 0.985*** |
|  |  | (5.855) | (6.324) |
| N | 406 | 406 | 406 |
| AIC | 1160.971 | 992.981 | 1014.029 |
| BIC | 1176.996 | 1017.019 | 1038.067 |
| t statistics in parentheses |  |  |  |
| * p<0.05; ** p<0.01; *** p<0.001 | |  |  |

| **Table D. Structural equation model parameter estimates for growth curves of IUD Satisfaction (5-level ordinal Likert scale); Multiple imputation, 30 imputations** | | | |
| --- | --- | --- | --- |
|  | IUD Satisfaction | IUD Satisfaction | IUD Satisfaction |
| Link Function | Linear | Ordered logistic | Ordered probit |
| Time (study month) | 0.064* | 0.154** | 0.100** |
|  | (2.450) | (2.652) | (3.019) |
| Intercept | 3.649*** |  |  |
|  | (33.150) |  |  |
| Random intercept variance | 0.478*** | 3.458*** | 0.940*** |
|  | (4.258) | (3.839) | (3.869) |
| Residual variance | 0.810*** |  |  |
|  | (12.137) |  |  |
| Threshold 1 |  | -3.529*** | -1.802*** |
|  |  | (-9.504) | (-9.923) |
| Threshold 2 |  | -2.548*** | -1.322*** |
|  |  | (-7.725) | (-7.802) |
| Threshold 3 |  | -1.087*** | -0.549*** |
|  |  | (-3.716) | (-3.513) |
| Threshold 4 |  | 1.637*** | 0.945*** |
|  |  | (5.529) | (5.924) |
| N_j_ (Assessments) | 462 | 462 | 462 |
| N_i_ (Subjects) | 77 | 77 | 77 |
| t statistics in parentheses |  |  |  |
| * p<0.05; ** p<0.01; *** p<0.001 | |  |  |

| **Table E. Structural equation model parameter estimates for growth curves of cramping (6-level ordinal MSQ item); Unimputed, complete case analysis** | | | |
| --- | --- | --- | --- |
|  | Cramping | Cramping | Cramping |
| Link Function | Linear | Ordered logistic | Ordered probit |
| Time (study month) | -0.133*** | -0.297*** | -0.179*** |
|  | (-5.443) | (-4.799) | (-5.183) |
| Intercept | 2.335*** |  |  |
|  | (19.783) |  |  |
| Random intercept variance | 0.698*** | 4.493*** | 1.292*** |
|  | (5.102) | (4.334) | (4.439) |
| Residual variance | 0.682*** |  |  |
|  | (12.916) |  |  |
| Threshold 1 |  | -3.832*** | -2.142*** |
|  |  | (-10.059) | (-10.750) |
| Threshold 2 |  | -2.464*** | -1.396*** |
|  |  | (-7.243) | (-7.660) |
| Threshold 3 |  | 1.083*** | 0.583*** |
|  |  | (3.449) | (3.418) |
| Threshold 4 |  | 2.273*** | 1.241*** |
|  |  | (6.771) | (6.948) |
| Threshold 5 |  | 4.947*** | 2.630*** |
|  |  | (9.363) | (10.158) |
| N | 412 | 412 | 412 |
| AIC | 1160.966 | 1051.237 | 1055.422 |
| BIC | 1177.050 | 1079.384 | 1083.570 |
| t statistics in parentheses |  |  |  |
| * p<0.05; ** p<0.01; *** p<0.001 |  |  |  |

| **Table F. Structural equation model parameter estimates for growth curves of cramping (6-level ordinal MSQ item); Multiple imputation, 30 imputations** | | | |
| --- | --- | --- | --- |
|  | Cramping | Cramping | Cramping |
| Link Function | Linear | Ordered logistic | Ordered probit |
| Time (study month) | -0.125*** | -0.280*** | -0.165*** |
|  | (-4.875) | (-4.378) | (-4.581) |
| Intercept | 2.324*** |  |  |
|  | (19.116) |  |  |
| Random intercept variance | 0.737*** | 4.474*** | 1.311*** |
|  | (4.997) | (4.203) | (4.237) |
| Residual variance | 0.713*** |  |  |
|  | (11.305) |  |  |
| Threshold 1 |  | -3.709*** | -2.077*** |
|  |  | (-9.743) | (-10.233) |
| Threshold 2 |  | -2.354*** | -1.334*** |
|  |  | (-6.893) | (-7.173) |
| Threshold 3 |  | 1.072*** | 0.587*** |
|  |  | (3.426) | (3.428) |
| Threshold 4 |  | 2.242*** | 1.235*** |
|  |  | (6.703) | (6.869) |
| Threshold 5 |  | 4.716*** | 2.523*** |
|  |  | (9.122) | (9.572) |
| N_j_ (Assessments) | 462 | 462 | 462 |
| N_i_ (Subjects) | 77 | 77 | 77 |
| t statistics in parentheses |  |  |  |
| * p<0.05; ** p<0.01; *** p<0.001 | |  |  |

| **Table G: IUD Satisfaction predicted by bleeding during study and cramping; Multiple imputation, 30 imputations** | | | |
| --- | --- | --- | --- |
| Outcome: | IUD Satisfaction | IUD Satisfaction |  |
|  | (latent mean) | (study end) |  |
| Link Function: | Linear | Ordered logistic |  |
| IUD-induced bleeding | -0.940** | -1.563** |  |
|  | (-2.663) | (-3.188) |  |
| Cramping | -0.033 | -0.082 |  |
|  | (-0.352) | (-0.759) |  |
| Intercept | -0.001 |  |  |
|  | (-0.003) |  |  |
| Threshold 1 |  | -4.626*** |  |
|  |  | (-4.544) |  |
| Threshold 2 |  | -2.717*** |  |
|  |  | (-6.075) |  |
| Threshold 3 |  | -0.895*** |  |
|  |  | (-3.412) |  |
| Threshold 4 |  | 0.571* |  |
|  |  | (2.286) |  |
| N | 77 | 77 |  |
| R-sq | 0.09 |  |  |
| Pseudo R-sq |  | 0.065 |  |
| t statistics in parentheses | |  |  |
| * p<0.05; ** p<0.01; *** p<0.001 | |  |  |

**Table H. SEM parameter estimates for growth curve trajectories for bleeding including the three retrospective baseline bleeding reports**

|  | | (A) Bleeding | | |  | |  |  |
| --- | --- | --- | --- | --- | --- | --- | --- | --- |
| *Coefficients (fixed effects)* | | Est | | SE | T-stat | | P-val | |
| Baseline (intercept) | | 122.5 | | 14.72 | 8.32 | | 0.000 | |
| Accelerated change (time) | | -0.1 | | 0.03 | -3.00 | | 0.003 | |
| IUD-induced bleeding | | 60.1 | | 11.86 | 5.07 | | 0.000 | |
| *Between-subject variance*  *(random effects)* | |  | |  |  | |  | |
| Baseline (intercept) | | 16126.7 | | 2694.9 | 5.98 | | 0.000 | |
| IUD-induced bleeding | | 4191.4 | | 1299.1 | 3.23 | | 0.001 | |
| Covariance(base,IUD) | | -6340.6 | | 1624.6 | -3.90 | | 0.000 | |

**Fig I. Time series plots summarizing distributions, and predicted mean growth curves of bleeding as measured by the Pictorial Blood Assessment Chart** (**PBAC), over study month, including the three retrospective baseline bleeding reports (i.e., months 1-3)**

Black diamonds represent PBAC variable means by month. Interval bars represent PBAC means +/- 0.5 PBAC SDs, by month. The red lines represent the model-implied growth curve as predicted by the SEM model summarized above in S8 Table. The underlying dotplot depicts the distributions of PBAC, by month. Values are binned, and the Y axis is top coded at ≥400 for the PBAC score to optimize resolution in the majority of the distribution.
